# Hypoxia-Related lncRNA Correlates With Prognosis and Immune Microenvironment in Lower-Grade Glioma

**DOI:** 10.3389/fimmu.2021.731048

**Published:** 2021-09-30

**Authors:** Shengchao Xu, Lu Tang, Zhixiong Liu, Chengke Luo, Quan Cheng

**Affiliations:** ^1^ Department of Neurosurgery, Xiangya Hospital, Central South University, Changsha, China; ^2^ Department of Thoracic Surgery, Xiangya Hospital, Central South University, Changsha, China; ^3^ National Clinical Research Center for Geriatric Disorders, Xiangya Hospital, Central South University, Changsha, China

**Keywords:** lower-grade glioma, hypoxia, long non-coding RNA, immune microenvironment, immune infiltration

## Abstract

**Background:**

Hypoxia-related genes are demonstrated to correlate with the prognosis of various cancers. However, the role of hypoxia-related long non-coding RNAs (HRLs) in lower-grade glioma (LGG) remains unclear.

**Methods:**

A total of 700 LGG samples were extracted from TCGA and CGGA databases. Pearson correlation analysis was used to identify HRLs. Lasso analysis was adopted to construct the HRL signature. TIDE algorithm was used to predict responses to immune checkpoint inhibitors. Cell proliferation was estimated by cell counting kit-8 assay, colony formation assay, and EdU assay.

**Results:**

We identified 340 HRLs and constructed a novel risk signature composed of 19 HRLs. The risk score exhibited potent value in predicting the prognosis of LGG patients and was significantly associated with the prognosis of LGG patients. Moreover, HRL signature could distinguish patients with similar expression levels of immune checkpoints and might predict the efficacy of immune checkpoint inhibitors. Additionally, hypoxia-related pathways and immune pathways were enriched in high-risk group, and high risk score indicated low tumor purity and high immune infiltration. Two major HRLs, LINC00941 and BASP1-AS1, could significantly affect the proliferation of glioma cells.

**Conclusions:**

Our study constructed a novel HRL signature that could predict the prognosis and immunotherapy response of LGG patients. HRLs could be novel biomarkers to predict the prognosis of LGG patients and potential targets for LGG treatment.

## Introduction

Glioma is the most common type of brain cancer (1). The World Health Organization (WHO) has classified gliomas into four grades, in which the higher grade indicates the higher malignancy. Patients with grade II glioma have a median overall survival (OS) of about 11 years, and those with grade III glioma have a median OS of approximately 3 years (2, 3). Since glioblastoma (grade IV glioma) has a superior malignancy and a poor prognosis, the Cancer Genome Atlas (TCGA) classified grade II and III gliomas as lower-grade glioma (LGG). Although great progress has been made to develop novel therapeutics against cancer, few drugs have been approved for LGG treatment, and the prognosis of LGG patients remains poor (4). Therefore, there is a clear urgent to develop novel biomarkers to predict the prognosis of LGG patients and find potential targets for the treatment of LGG.

Hypoxia has been implicated to promote the progression of tumors with the induction of hypoxic tumor context (5). The occurrence and development of tumors often accompanies with several adaptive alternations such as angiogenesis, proliferation, and so on, where hypoxia can promote the aggressiveness of tumors (6). In glioblastoma, extensive tissue hypoxia is commonly detected, and it can facilitate the formation of glioma stem-like cells, which is closely associated with tumor recurrence (7). Besides, hypoxia-related genes are demonstrated to correlate with the prognosis of glioma patients (8, 9). Therefore, hypoxia is crucial for glioma development.

In recent years, long non-coding RNA (lncRNA) have emerged to play diverse roles in various biological processes (10). These lncRNAs can modulate transcriptional and post-transcriptional of genes and regulate the expression of tumor suppressors or initiators, which confers the occurrence and progression of cancer (11). In gliomas, lncRNA has been implicated to be associated with the proliferation, invasion, and prognosis of glioma cells (12–15). However, no research has comprehensively revealed the role of hypoxia-related lncRNAs (HRLs) in LGG.

A previous study indicated that hypoxia-related signature was associated with the prognosis and immune microenvironment of glioma patients (8). However, the role of HRLs in LGG remained unclear. Herein, our study extracted data from TCGA and Chinese Glioma Genome Atlas (CGGA) databases to identify candidate HRLs and constructed related signature, aiming to explore its prognostic value in LGG patients and its association with LGG immune microenvironment.

## Materials and Methods

### Data Extraction

A total of 700 grade II and III glioma samples were included in our study. The RNA-seq and clinical data were extracted from CGGA (http://www.cgga.org.cn/) and TCGA (https://portal.gdc.cancer.gov/) databases. In this study, TCGA-LGG dataset (n=522) was defined as the training cohort, whereas CGGA dataset (n=178) was the validation cohort. The characteristics of glioma samples in this study were summarized in **Table 1**. Moreover, IMvigor210 dataset, a cohort of atezolizumab (anti-PD-L1 monoclonal antibody) for the treatment of urothelial carcinoma, was extracted to evaluate the predictive value of HRL signature for the efficacy of immunotherapy (16).

**Table 1:** Characteristics of the training cohort and validation cohort.

Features	Training cohort	Validation cohort
	TCGA (n=522)	CGGA (n=178)
Age		
≤ 45	320	135
> 45	202	43
Gender		
Male	289	107
Female	233	71
Grade		
II	256	102
III	265	76
NA	1	0
IDH status		
Mutant	423	131
Wildtype	96	47
NA	3	0
1p/19q status		
Codel	171	60
Non-codel	351	118
MGMT status		
Unmethylated	91	86
Methylated	431	76
NA	0	16

IDH, isocitrate dehydrogenase; MGMT, O6 -methylguanine-DNA methyltransferase.

### Identification of HRLs

The 26 hypoxia-related genes were reported by previous studies (17, 18). A total of 14,488 and 13,895 lncRNAs were identified in the TCGA and CGGA datasets, respectively. Those lncRNAs whose expressions closely corelated with the expression of 26 hypoxia-related genes (|R|>0.5 and p<0.01) were identified as HRLs.

### Bioinformatic Analyses

To evaluate the involvement of biological processes of each sample, gene set variation analysis (GSVA) was conducted to quantify the involvement of Gene Ontology pathways in each LGG sample (19). As for gene set enrichment analysis (GSEA), differentially expressed genes between two groups were identified. Those with false discovery rate (FDR) ≤0.05 were selected for GSEA analysis. Gene sets of hallmarks were obtained from the Molecular Signatures Database (MSigDB). Gene Ontology (GO) and Kyoto Encyclopedia of Genes and Genomes (KEGG) enrichment analyses were conducted by “clusterprofiler” R package. The nomogram and calibration curves were constructed and visualized using “rms” and “regplot” R packages. Tumor Immune Dysfunction and Exclusion (TIDE) algorithm was used to predict the responses of glioma patients to immune checkpoint inhibitors (ICIs) (20).

### Estimation of Immune Microenvironment

Estimation of Stromal and Immune cells in Malignant Tumor tissues using Expression data (ESTIMATE) analysis was conducted to calculate the tumor purity of each sample by “estimate” R package (21). The infiltration of immune cells was estimated by single-sample Gene Set Enrichment Analysis (ssGSEA) and Tumor Immune Estimation Resource (TIMER), and Cell-type Identification by Estimating Relative Subsets of RNA Transcripts (CIBERSORT) algorithms (22–24).

### Construction of Risk Signature

The risk signature was constructed using the least absolute shrinkage and selection operator (LASSO) analysis. The risk score was calculated by the following algorithm:


$$\text{Risk Score}=\Sigma_{i=1}^{n}Coefficient_{\text{i}}*Gene_{\text{i}}$$

### Subgroup Analysis

For subgroup analysis, LGG patients were divided into different groups based on the following variables: grade (grades II or III), age (≤45 years old or >45 years old), and IDH status (mutant or wildtype).

### Construction of Competing Endogenous RNA Network

The potential target miRNAs of LINC00941 and BASP1-AS1 were predicted using ENCORI online webtool (http://starbase.sysu.edu.cn/). Then, the potential target mRNAs of these miRNAs were predicted using microT-CDS (http://diana.imis.athena-innovation.gr/DianaTools/index.php?r=microT_CDS/index) and mirDIP online webtools (http://ophid.utoronto.ca/mirDIP/). Pearson correlation analysis was conducted to screen genes co-expressed with LINC00941 or BASP1-AS1 in the TCGA dataset with r>0.4 and p<0.001. These genes were intersected with predicted mRNAs to define potential mRNA targets of LINC00941 or BASP1-AS1. Finally, the lncRNA-miRNA-mRNA network was constructed using Cytoscape 3.8.0.

### Cell Culture and Cell Transfection

Given the fact that there were no widely used LGG cell lines, and glioblastoma and LGG belonged to gliomas, we used two glioblastoma cells (DBTRG-05MG and U251 MG) in this study to explore the effect of HRLs on gliomas. DBTRG was cultured in RPMI-1640 medium with 10% fetal bovine serum (FBS, ExCell Bio, China), and U251 was cultured in DMEM medium with 10% FBS (ExCell Bio, China). Specific siRNAs targeting LINC00941 and BASP1-AS1 were designed and synthesized by GenePharma. Lipofectamine 3000 (Invitrogen, USA) was used for cell transfection.

### Extraction of Total RNA and Quantitative Real-Time PCR

Total RNA was extracted using TRIzol reagent (Invitrogen, USA). The PrimeScript RT reagent Kit (RR047A, Takara) was used to synthesize cDNA. The TB Green Fast qPCR Mix (RR430S, Takara) was used for qPCR. GAPDH was used as the reference gene. Primers used in this study were as follows: GAPDH: F, CAGGAGGCATTGCTGATGAT; R, GAAGGCTGGGGCTCATTT. LINC00941: F, ACCACTACACTCAGCCAAATAC; R, GGCTATCAACTGTCTCCTTTAGAC. BASP1-AS1: F, AGCACCGGGACACAGAATAG; R, TTTGCGGGAAGGTAAAATTG.

### Cell Counting Kit-8 Assay

In each well of 96-well plate, 2×10^3^ cells were inoculated and maintained in culture medium for 0, 24, 48, and 72 h. Then, 10 μl CCK-8 reagent (DOJINDO, Japan) was added into each well, and the optical density of 450 nm was estimated.

### Colony Formation Assay

In each well of six-well plate, 800 cells were seeded and maintained in culture medium for 14 days. After the fix with 4% paraformaldehyde, cells were stained with 0.1% crystal violet.

### 5-Ethynyl-2’-Deoxyuridine Assay

A total of 1×10^5^ cells was seeded in 20 mm round coverslip. The EdU Cell Proliferation Assay Kit (Ribobio, China) was used for EdU assay and the processes were conducted according to the manufacturer’s instructions.

### Statistical Analysis

Statistical analyses and visualization were mainly performed using R version 3.6.0 and GraphPad Prism version 8.0.1. Student’s t test and one-way ANOVA analysis were used to estimate the differences between two groups and more than two groups. Kaplan-Meier analysis was conducted to compare the survival differences between two groups of patients. Multivariate Cox analysis was used to evaluate the prognostic value of risk score. The correlation of gene expression was determined by Pearson correlation analysis. Time-dependent receiver operating characteristic (ROC) curve analysis was adopted to estimate the predictive value of risk score. Two-sided p ≤ 0.05 was regarded as statistically significant.

## Results

### Identification of HRLs in the TCGA and CGGA Datasets

**Figure 1:**
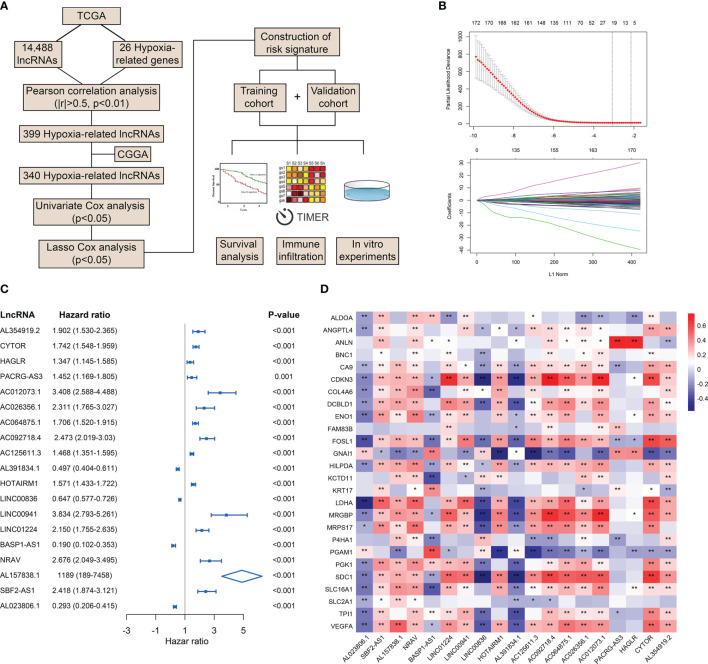
Identification of HRLs in the TCGA and CGGA datasets. **(A)** Flow chart of the whole study. **(B)** Lasso analysis of prognostic HRLs with minimum lambda value. **(C)** Univariate Cox analysis of 19 selected HRLs. **(D)** Heatmap of the correlation between hypoxia-related genes and HRLs. *p < 0.05, **p < 0.01.

To preliminarily explore the role of hypoxia and lncRNAs in LGG, we screened HRLs in the TCGA dataset. A total of 14,488 lncRNAs and 26 hypoxia-related genes were identified and selected for our study (**Figure 1A**). Pearson correlation analysis identified 399 HRLs (|R|>0.5 and p<0.01) in the TCGA dataset (**Table S1**). After the intersection with lncRNAs in the CGGA dataset, 340 HRLs were identified. With the application of univariate Cox analysis and Lasso analysis, 19 HRLs were selected for further analysis (**Figure 1B**). Among these lncRNAs, four lncRNAs (AL391834.1, LINC00836, BASP1-AS1, and AL023806.1) played protective roles in LGG, whereas the other 15 lncRNAs were risk factors (p<0.05) (**Figure 1C**). Kaplan-Meier analysis further validated the prognostic values of these lncRNAs in the two datasets (p<0.05) (**Figure S1**). The correlation between 19 selected HRLs and 26 hypoxia-related genes were shown by the heatmap (**Figure 1D**). These results indicated that identified lncRNAs were correlated with hypoxia and were prognostic biomarkers of LGG.

### Risk Signature Based on HRL Correlated With the Prognosis and Clinical Features in LGG

**Figure 2:**
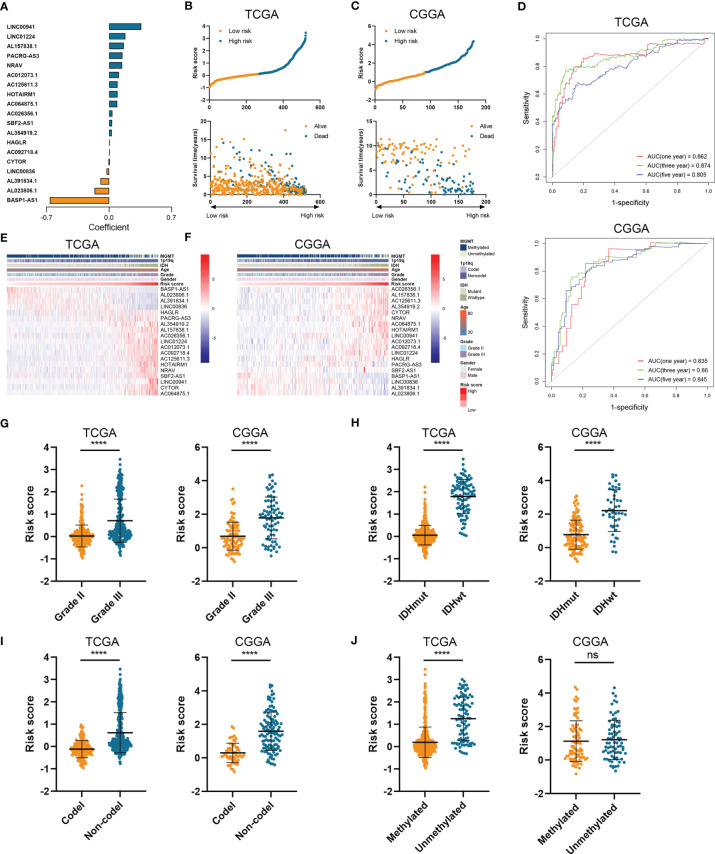
Construction of risk signature based on the expression of HRLs. **(A)** The coefficient of 19 HRLs for the construction of risk signature. **(B, C)** The risk score and survival time of each sample in the TCGA **(B)** and CGGA **(C)** datasets. **(D)** Time-dependent ROC analysis of risk score in predicting 1-, 3-, and 5-year survival. **(E, F)** The expression of 19 HRLs in each sample from low risk to high risk in the TCGA **(E)** and CGGA **(F)** datasets. **(G–J)** The risk score in different grades **(G)**, IDH status **(H)**, 1p19q status **(I)**, and MGMT status **(J)** of gliomas in the two datasets. ****p < 0.0001; ns, no significance.

Then we constructed the HRL signature to further characterize the role of HRLs in LGG. With the application of Lasso analysis, the coefficient of 19 HRLs was determined, in which the coefficient of four lncRNAs (AL391834.1, LINC00836, BASP1-AS1, and AL023806.1) was negative and that of the other lncRNAs was positive (**Figure 2A**). The risk score of each LGG patients was calculated according to the coefficient and expression of 19 HRLs. Risk score was significantly associated with the expression of hypoxia-related genes (**Figure S2**). Then, LGG patients were divided into high-risk and low-risk groups based on the medium value of risk score (**Figures 2B, C**). Patients whose survival time was relatively short and status was censored were enriched in high-risk group (**Figures 2B, C**). The area under curve (AUC) of risk score in predicting 1-, 3-, and 5-year survival of LGG patients was 0.862, 0.874, and 0.805, respectively, in the TCGA dataset, whereas those were 0.835, 0.860, and 0.845, respectively, in the CGGA dataset (**Figure 2D**). Four protective lncRNAs (AL391834.1, LINC00836, BASP1-AS1, and AL023806.1) were highly expressed in low-risk group, whereas other lncRNAs were highly expressed in high-risk group (**Figures 2E, F**). Regarding several well-known biomarkers of glioma, risk score was significantly elevated in grade III glioma compared with grade II one (p<0.05) (**Figure 2G**). In the meantime, risk score was significantly lower in IDH mutant and 1p19q co-deleted gliomas (p<0.05) (**Figures 2H, I**). However, as for the methylation of MGMT promoter, risk score did not share the consistent trend in the TCGA and CCGA dataset, where risk score was significantly elevated in MGMT unmethylated glioma in the TCGA dataset (p<0.05) but not in CCGA dataset (p>0.05) (**Figure 2J**). These findings suggested that the constructed risk signature based on the expression of HRLs was associated with clinical features of LGG and could predict the survival time of LGG patients.

### Risk Score Was an Independent Risk Factor for LGG Patients

**Figure 3:**
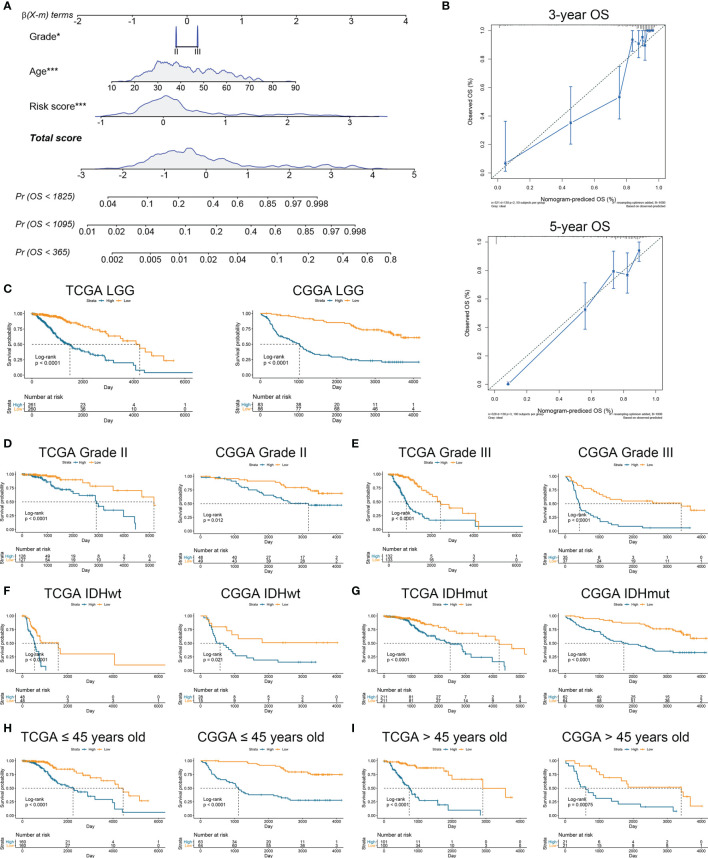
Risk signature was associated with the prognosis of LGG patients. **(A)** Nomogram model of grade, age, and risk score in the TCGA dataset. **(B)** Calibration curve model to verify the predictive value of risk score regarding 3-year and 5-year survival. **(C)** Kaplan-Meier analysis of high-score and low-score in LGG patients. **(D, E)** Kaplan-Meier analysis of high-score and low-score patients in grade II **(D)** and grade III **(E)** gliomas. **(F, G)** Kaplan-Meier analysis of high-score and low-score patients in IDH wildtype **(F)** and IDH mutant **(G)** gliomas. **(H, I)** Kaplan-Meier analysis of high-score and low-score patients ≤45 years old **(H)** or >45 years old **(I)** in diffuse gliomas.

To further verify the prognostic value of risk signature, we constructed a nomogram model, whose C-indexes were 0.857 and 0.772 in the TCGA and CGGA datasets, respectively (**Figure 3A**). Moreover, a calibration plot for probability of survival exhibited satisfactory concordance with the prediction of 3-year and 5-year OS in the TCGA dataset (**Figure 3B**). Then we conducted a subgroup analysis to verify the prognostic value of HRL signature in different subgroups of LGG patients. Results showed that LGG patients with high risk score had poor prognosis (p<0.05) (**Figure 3C**). As for grade II and III gliomas, high risk score indicated worse prognosis (p<0.05) (**Figures 3D, E**). Similarly, in IDH mutant or wildtype LGG patients, those in low-risk group had longer survival time (p<0.05) (**Figures 3F, G**). Moreover, when patients were divided into young (age ≤45 years old) and old (age >45 years old) groups, the prognostic value of risk score was consistent (p<0.05) (**Figures 3H, I**). Furthermore, multivariate Cox analysis revealed that risk score and grade were independent risk factors for LGG patients in the TCGA and CGGA datasets (p<0.05) (**Table 2**). These results indicated that risk score was a potent marker to predict the prognosis of LGG patients.

**Table 2:** Multivariate analysis of risk signature in the training and validation cohorts.

Variables	TCGA (n=522)		CGGA (n=178)	
	HR (95% CI)	P value	HR (95% CI)	P value
Risk score	4.093 (2.884–5.810)	<0.001	1.554 (1.197–2.018)	0.001
Age	1.051 (1.033–1.068)	<0.001	1.011 (0.988–1.034)	0.351
Gender	1.221 (0.850–1.756)	0.280	0.595 (0.370–0.956)	0.032
Grade	1.491 (0.962–2.312)	0.004	2.755 (1.640–4.628)	<0.001
IDH	0.405 (0.193–0.852)	0.017	0.656 (0.353–1.220)	0.183
1p19q	1.574 (0.919–2.695)	0.099	3.825 (1.835–7.972)	<0.001
MGMT	1.028 (0.640–1.649)	0.910	1.325 (0.802–2.190)	0.272

HR, hazard ratio; CI, confidence interval; IDH, isocitrate dehydrogenase; MGMT, O6 -methylguanine-DNA methyltransferase.

### Risk Stratification Correlated With the Efficacy of Immunotherapy

**Figure 4:**
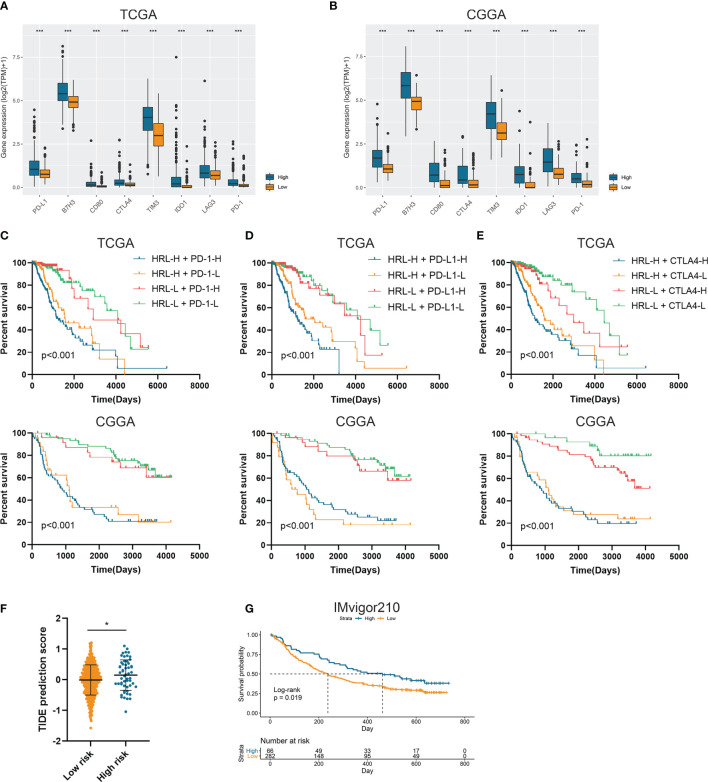
Risk stratification correlated with the efficacy of immunotherapy. **(A, B)** The expression of several immune checkpoints in high-risk and low-risk groups in the TCGA **(A)** and CGGA **(B)** datasets. **(C–E)** Kaplan-Meier analyses of overall survival among four patient groups stratified by the HRL signature and PD-1 **(C)**, PD-L1 **(D)**, and CTLA-4 **(E)**. **(F)** TIDE score of high-risk and low-risk groups in TCGA dataset. **(G)** Kaplan-Meier analysis of urothelial carcinoma patients stratified by HRL signature in IMvigor210 cohort. *p < 0.05, ***p < 0.001.

Since immunotherapy was a promising therapeutic approach in cancer treatment, we explored the association between risk stratification and the efficacy of ICIs. The expression of several immune checkpoints including PD-1, PD-L1, CTLA-4, TIM-3, B7-H3, IDO1, and LAG3 was significantly elevated in high-risk group compared with the low-risk group in the TCGA and CGGA datasets (p<0.05) (**Figures 4A, B**). Patients with low risk score and low PD-1 expression had significantly better prognosis than those with high risk score and low PD-1 expression (p<0.05) (**Figure 4C**), and patients with low risk score and high PD-1 had prolonged survival than those with high risk score and high PD-1 (p<0.05) (**Figure 4C**). Similarly, the stratification based on HRL signature and immune checkpoints was associated with significant survival difference in LGG patients. Patients with low risk score tended to have better prognosis no matter when the immune checkpoints (PD-L1 and CTLA-4) were highly or lowly expressed (p<0.05) (**Figures 4D, E**). With the application of TIDE algorithm, we found that TIDE score was significantly elevated in high-risk group, which indicated that patients in high-risk group had worse responses to immunotherapy compared with low-risk group (p<0.05) (**Figure 4F**). Thereafter, we extracted the data from IMvigor210 dataset, a cohort of atezolizumab for the treatment of urothelial carcinoma, to investigate the correlation between HRL signature and immunotherapeutic efficacy. Due to the limited number of lncRNA in IMvigor210 dataset, only 11 out of 19 HRLs were identified, and risk score was calculated based on the coefficient and expression of 11 HRLs. Results showed that HRL signature was significantly correlated with the survival of urothelial carcinoma patients receiving atezolizumab treatment (p<0.05) (**Figure 4G**). However, in the IMvigor210 cohort, patients with high risk score had a better prognosis, which might be due to the different roles of HRLs in different types of cancer. Nevertheless, these findings suggested that HRL signature might be a potential biomarker to predict the therapeutic response of immune checkpoint inhibitors.

### High-Risk Group Exhibited Distinct Immune Characteristics

**Figure 5:**
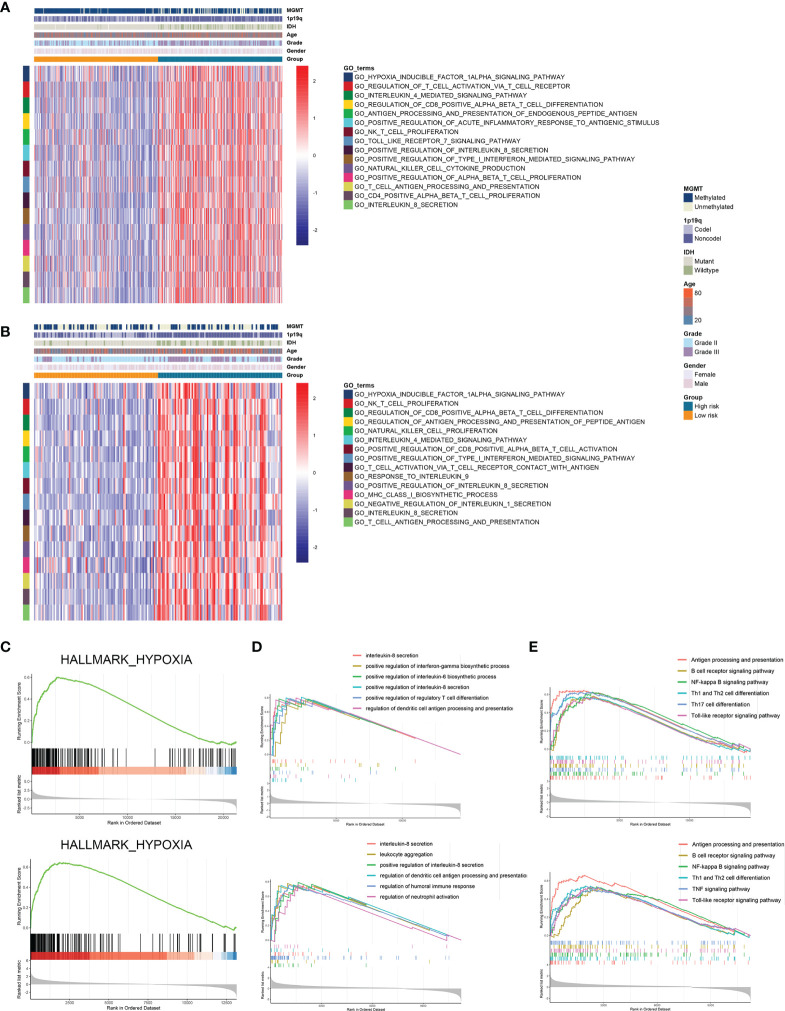
Risk stratification exhibited distinct immune characteristics. **(A, B)** GSVA analysis of hypoxia and immune pathways in high-risk and low-risk groups in the TCGA **(A)** and CGGA **(B)** datasets. **(C)** Differentially expressed genes between high-risk and low-risk groups were enriched in hypoxia-related pathways. **(D, E)** GSEA analysis of differentially expressed genes between high-risk and low-risk groups in GO **(D)** and KEGG **(E)** terms.

Further we explored the potential pathways that were associated with the prognosis of patients in high-risk and low-risk groups. GSVA analysis revealed that hypoxia inducible factor 1 (HIF-1) signaling pathway and immune-related pathways including lymphocyte activation, interleukin-mediated signaling pathway, and antigen processing and presentation were highly enriched in high-risk group (**Figures 5A, B**). Differentially expressed genes between high-risk and low-risk groups were screened in the TCGA and CGGA datasets (**Figure S3**). GSEA analysis showed that genes highly expressed in the high-risk group were enriched in hypoxia-related pathway (**Figure 5C**). Meanwhile, these genes were involved in antigen processing and presentation as well as interleukin secretion in GO pathways (**Figure 5D**); in KEGG terms, these genes were associated with antigen processing and presentation, T cell differentiation, and B cell receptor signaling pathway (**Figure 5E**). Therefore, risk stratification based on risk scores was associated with hypoxia-related pathways, and the high-risk group exhibited highly activated immune characteristics.

### High Risk Score Indicated Low Tumor Purity and High Immune Infiltration

**Figure 6:**
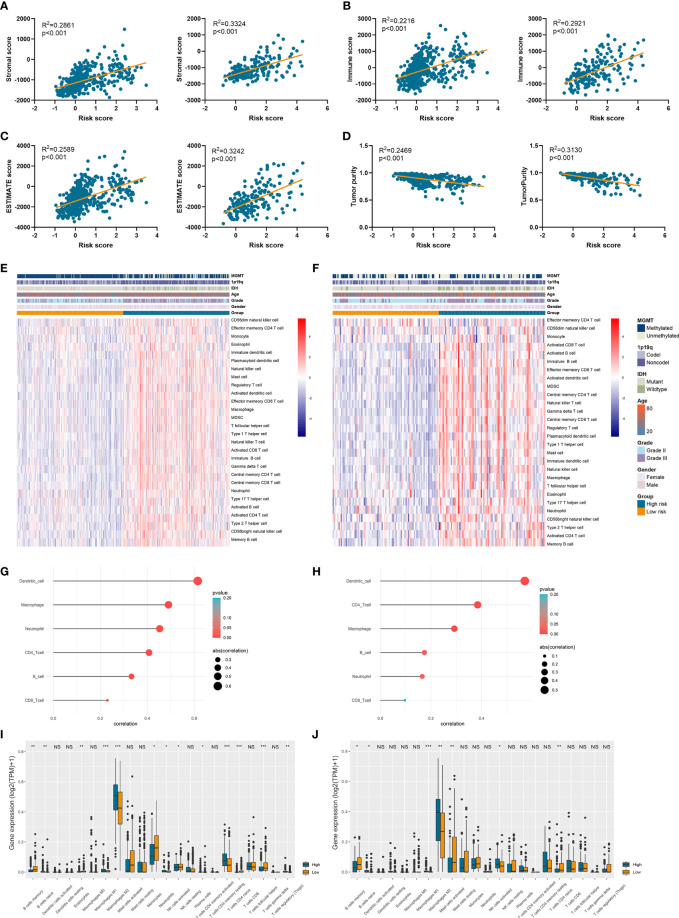
Risk signature was associated with tumor purity and immune infiltration. **(A–D)** The association between risk score and stromal score **(A)**, immune score **(B)**, ESTIMATE score **(C)**, and tumor purity **(D)**. **(E, F)** The abundance of 28 immune cells estimated by ssGSEA algorithm in high-risk and low-risk groups in TCGA **(E)** and CGGA **(F)** datasets. **(G, H)** The correlation between risk score and six immune cells estimated by TIMER algorithm in TCGA **(G)** and CGGA **(H)** datasets. **(I, J)** The abundance of 22 immune cells estimated by CIBERSORT algorithm in high-risk and low-risk groups in TCGA **(I)** and CGGA **(J)** datasets. *p < 0.05, **p < 0.01, ***p < 0.001, ns, no significance.

Since tumor immune microenvironment was implicated to be associated with the prognosis of LGG patients (25, 26), we also explored the correlation between risk signature and immune microenvironment in LGG. The risk score was significantly positively associated with the stromal score, immune score, and ESTIMATE score (p<0.05) (**Figures 6A–C**). Then tumor purity was calculated according to the algorithm based on ESTIMATE score (21), and high risk score notably indicated low tumor purity (p<0.05) (**Figure 6D**). The infiltration of immune cells was estimated by the conduct of ssGSEA and TIMER algorithms, which contained 28 and six immune cells, respectively. Immune cells such as macrophages, activated T cells, activated B cells, and activated dendritic cells were enriched in high-risk group of samples (**Figures 6E, F**). Besides, risk score was significantly correlated with the abundance of dendritic cell, macrophage, and CD4+ T cells (p<0.05) (**Figures 6G, H**). In addition, CIBERSORT algorithm revealed that the abundance of B cells, macrophages (M1 and M2 subtypes), and naïve CD4+ T cells were highly infiltrated in high-risk group (p<0.05) (**Figures 6I, J**). These results suggested that risk signature was associated with immune infiltration, and high risk score implied low tumor purity, which might account for its risk role in the prognosis of LGG patients.

### Two Prognostic HRLs Were Associated With Prognosis and Immune Infiltration

**Figure 7:**
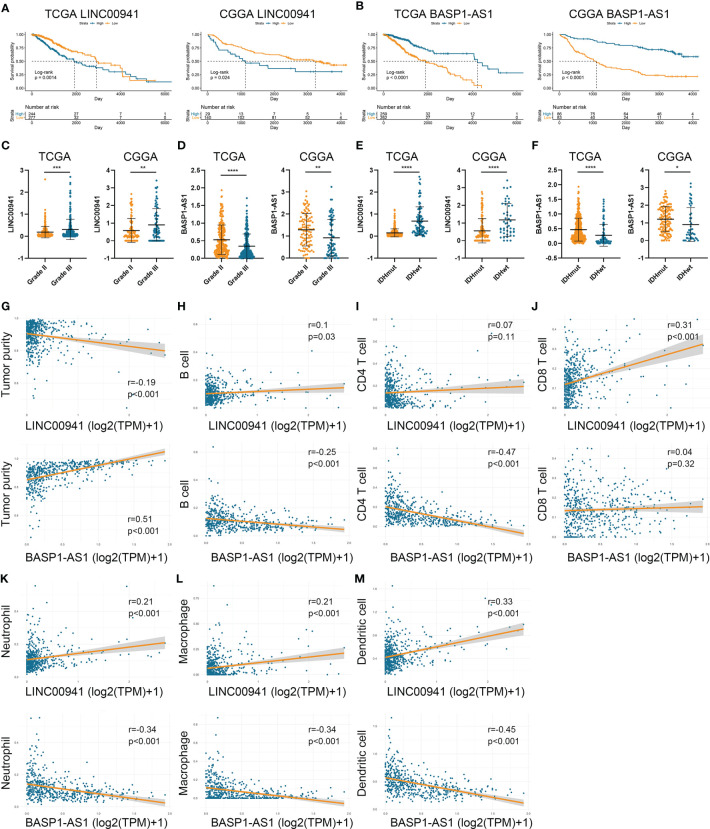
LINC00941 and BASP1-AS1 were associated with the prognosis and immune infiltration in LGG. **(A, B)** Kaplan-Meier analysis of LINC00941 and BASP1-AS1 in LGG patients of TCGA **(A)** and CGGA **(B)** datasets. **(C, D)** The expression of LINC00941 **(C)** and BASP1-AS1 **(D)** in grade II and III gliomas. **(E, F)** The expression of LINC00941 **(E)** and BASP1-AS1 **(F)** in IDH wildtype and IDH mutant gliomas. **(G)** The correlation between LINC00941 and BASP1-AS1 with tumor purity. **(H–M)** The correlation between LINC00941 and BASP1-AS1 with the abundance of B cell **(H)**, CD4+ T cell **(I)**, CD8+ T cell **(J)**, neutrophil **(K)**, macrophage **(L)**, and dendritic cell **(M)**. *p < 0.05, **p < 0.01, ***p < 0.001, ****p < 0.0001.

To investigate the role of HRLs in LGG, we selected two HRLs (LINC00941 and BASP1-AS1), which had the largest absolute coefficient in risk signature, for further analysis. In the TCGA and CGGA datasets, low expression of LINC00941 and high expression of BASP1-AS1 indicated favorable prognosis in LGG patients (p<0.05) (**Figures 7A, B**). Moreover, the expression of LINC00941 was significantly elevated in grade III glioma than grade II one, whereas that of BASP1-AS1 exhibited opposite expression pattern (p<0.05) (**Figures 7C, D**). Similarly, LINC00941 was highly expressed in IDH wildtype glioma, and BASP1-AS1 was highly expressed in IDH mutant glioma (p<0.05) (**Figures 7E, F**). Therefore, LINC00941 and BASP1-AS1 were significantly associated with the prognosis and clinical features of LGG patients. Besides, we found that LINC00941 was significantly negatively correlated with tumor purity (r=−0.19, p<0.05) whereas BASP1-AS1 was positively associated with tumor purity (r=0.51, p<0.05) (**Figure 7G**). LINC00941 was significantly positively associated with the infiltration of B cell, CD8+ T cell, neutrophil, macrophage, and dendritic cell (|r|>0.1, p<0.05), whereas BASP1-AS1 was negatively associated with the infiltration of B cell, CD4+ T cell, neutrophil, macrophage, and dendritic cell (|r|>0.25, p<0.05) (**Figures 7H–M**). Therefore, LINC00941 and BASP1-AS1 were significantly associated with the prognosis and immune infiltration in LGG.

### LINC00941 and BASP-AS1 Exerted Diverse Effects on the Proliferation of Glioma Cells

**Figure 8:**
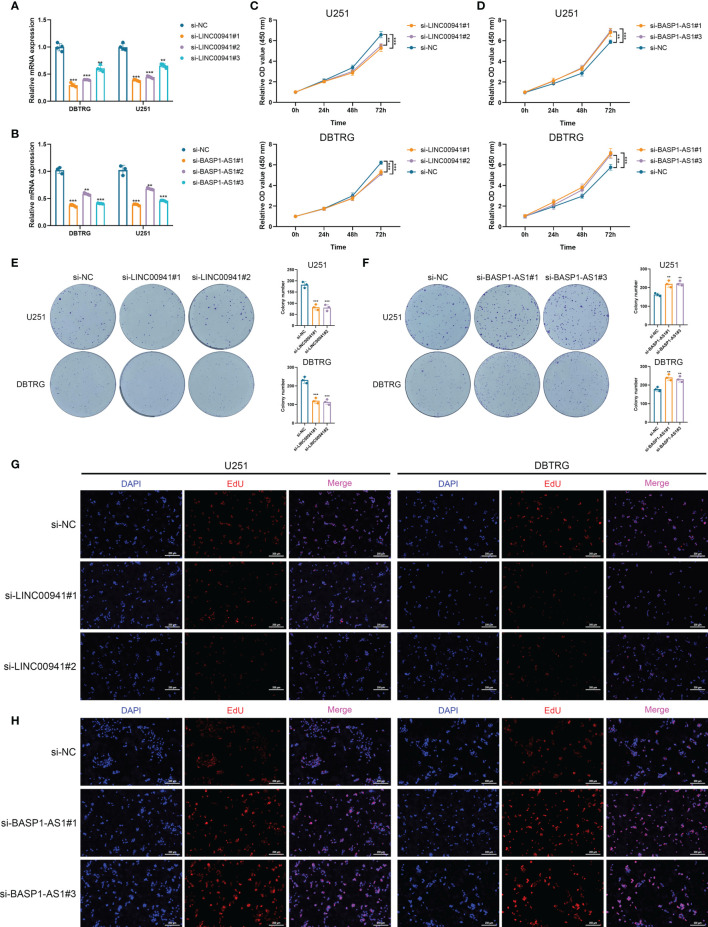
LINC00941 and BASP1-AS1 exerted opposite effects on the proliferation of glioma cells. **(A, B)** The expression of LINC00941 **(A)** and BASP1-AS1 **(B)** after the transfection of three specific siRNAs. **(C, D)** CCK-8 assay of U251 and DBTRG cells after the knockdown of LINC00941 **(C)** and BASP1-AS1 **(D)**. **(E, F)** Colony formation assay of U251 and DBTRG cells after the knockdown of LINC00941 **(E)** and BASP1-AS1 **(F)**. **(G, H)** EdU assay of U251 and DBTRG cells after the knockdown of LINC00941 **(G)** and BASP1-AS1 **(H)**. **p < 0.01, ***p < 0.001.

Then we performed *in vitro* experiments to verify the pathogenic role of LINC00941 and BASP1-AS1 in glioma cells. Three siRNAs were transfected in U251 and DBTRG cells to inhibit the expression of LINC00941 and BASP1-AS1, in which si-LINC00941#1 and si-LINC00941#2 as well as si-BASP1-AS1#1 and si-BASP1-AS1#3 were selected with the relatively high efficiency (p<0.05) (**Figures 8A, B**). The inhibition of LINC00941 significantly reduced the proliferation of glioma cells, whereas the inhibition of BASP1-AS1 significantly promoted their proliferation rates (p<0.05) (**Figures 8C, D**). Colony formation assay indicated that the knockdown of LINC00941 markedly decreased the colony number, whereas the knockdown of BASP1-AS1 exerted reversed effects (p<0.05) (**Figures 8E, F**). Moreover, EdU assay revealed that the proliferation of glioma cells was suppressed by the inhibition of LINC00941 and promoted by the inhibition of BASP1-AS1 (**Figures 8G, H**). Therefore, two prognostic HRLs, LINC00941 and BASP1-AS1, were associated with the proliferation of glioma cells and were potential therapeutic targets for glioma.

### Bioinformatic Analysis of Molecular Mechanisms Underlying LINC00941 and BASP1-AS1

**Figure 9:**
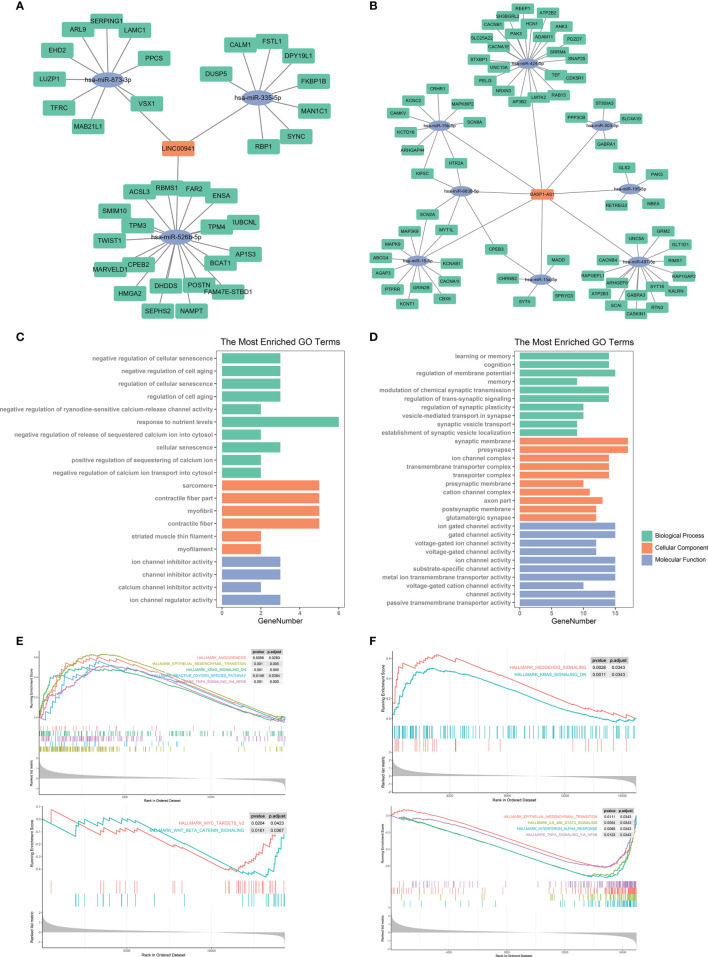
Bioinformatic analysis of molecular mechanisms underlying LINC00941 and BASP1-AS1. **(A, B)** Competing endogenous RNA network of LINC00941 **(A)** and BASP1-AS1 **(B)**. **(C, D)** Enrichment analysis of targeting mRNAs of LINC00941 **(C)** and BASP1-AS1 **(D)**. **(E, F)** Gene set enrichment analysis of differentially expressed genes between high- and low-expression of LINC00941 **(E)** and BASP1-AS1 **(F)**.

The discovery of ceRNA provided a novel insight into the pathogenic role of lncRNA in cancers. Therefore, we applied bioinformatic analyses to explore the potential miRNA and mRNA targets of LINC00941 and BASP1-AS1. Since lncRNA tended to elevate the expression of mRNA by acting as ceRNA, we screened co-expressed mRNAs of LINC00941 and BASP1-AS1 in the TCGA dataset. After the intersect with predicted miRNA and mRNA targets, the ceRNA network of LINC00941 and BASP1-AS1 was constructed (**Figures 9A, B**). Enrichment analysis revealed that LINC00941-targeting mRNAs were associated with cellular senescence and ion channel activity, whereas BASP1-AS1-targeting mRNAs were enriched in membrane potential, synaptic activity, and ion channel activity (**Figures 9C, D**). Differentially expressed genes were screened between high and low expression of LINC00941 or BASP1-AS1 groups (**Figures S3C, D**). GSEA revealed that epithelial mesenchymal transition (EMT), K-ras signaling, reactive oxygen species pathway, and TNF-α signaling pathway were highly enriched in high-LINC00941 group, whereas myc and Wnt/β-catenin were enriched in low-LINC00941 group (**Figure 9E**). Meanwhile, Hedgehog and K-ras signaling pathways were enriched in high-BASP1-AS1 group, and EMT, IL-6/JAK/STAT3, Interferon-α, and TNF-α signaling pathways were enriched in low-BASP1-AS1 group (**Figure 9F**). Therefore, these results indicated that LINC00941 and BASP1-AS1 might affect the proliferation of glioma cells by regulating ion channel activity *via* modulating EMT and TNF-α signaling pathway.

## Discussion

Hypoxia and lncRNA have been implicated to be critical factors to promote the progression of glioma (27, 28). In our study, we identified 340 HRLs and constructed a novel risk signature composed of 19 HRLs. The risk score exhibited potent value in predicting the prognosis of LGG patients and was significantly associated with the prognosis of LGG patients. Moreover, HRL signature could distinguish patients with similar expression levels of immune checkpoints and might predict the efficacy of ICIs. Additionally, hypoxia-related pathways and immune pathways were enriched in high-risk group, and high risk score indicated low tumor purity and high immune infiltration. *In vitro* experiments revealed that two major HRLs, LINC00941 and BASP1-AS1, could significantly affect the proliferation of glioma cells, in which EMT and TNF-α signaling pathway might be the underlying mechanism. Our study revealed a novel HRL signature to predict the prognosis of LGG patients. LINC00941 and BASP1-AS1 could be potential targets for LGG treatment.

Hypoxia microenvironment induced by tumor cells could facilitate the progression of tumors. Multiple studies revealed that hypoxia-related genes were associated with the prognosis of patients with pancreatic cancer, lung adenocarcinoma, head and neck cancer, bladder cancer, and other malignancies (18, 29–31). In high-risk bladder cancer, a signature composed of 24 hypoxia-related genes could significantly predict the prognosis and benefit from radiotherapy (31). Another 28-gene hypoxia signature also exhibited potent ability to predict the prognosis of bladder cancer patients (32). Moreover, a 26-gene hypoxia signature was shown to predict the benefit from hypoxia-modifying treatment in laryngeal cancer (17). When combination with immune genes, head and neck cancer patients were classified into three groups, in which those with low-hypoxia and high-immune characteristics had relatively favorable prognosis (18). In our study, 26 hypoxia-related genes were selected to screen HRLs, in which GNAI1 and PGAM1 were significantly associated with almost all HRLs. Kaplan-Meier analysis revealed that GNAI1 and PGAM1 were protective factors for LGG patients. Besides, GNAI1 and PGAM1 were positively associated with the expression of BASP1-AS1, whose high expression indicated favorable prognosis in LGG. Meanwhile, the expression of GNAI1 and PGAM1 was negatively associated with that of LINC00941 or LINC01224, which were risk factors for LGG patients. Therefore, GNAI1 and PCAM1 might be potential tumor suppressor in LGG. Further, we constructed a 19-lncRNA hypoxia signature and calculated risk score of LGG patients. Further analyses revealed that risk score had a potent accuracy in predicting the survival of LGG patients. Besides, high risk score indicated poor prognosis in different subgroups of LGG patients. When other clinical features were taken into consideration, risk score remained to be an independent risk factor for LGG patients. Therefore, our study revealed a novel HRL signature that could be used to predict the prognosis of LGG patients.

ICIs have shown promising efficacy in clinical care of various cancers (33, 34). However, since different patients exhibited diverse responses to ICIs, the discovery of predictive biomarkers would benefit cancer patients receiving ICIs. Although PD-L1 has been proposed to be a biomarker that is positively associated with the efficacy of ICI, the single biomarker is insufficient for cancer patients (35, 36). TIDE algorithm was developed by Jiang et al. to predict the responses to ICIs through characterizing dysfunctional T cells and infiltrated cytotoxic T lymphocytes (CTLs) level (20). In our study, we found that immune checkpoints were highly expressed in high-risk group stratified by HRL signature. HRL signature could distinguish patients with similar expression levels of immune checkpoints. Moreover, patients with low risk score and low immune checkpoints expression had significantly prolonged survival, which indicated that low risk score was associated with better response to ICIs. Since high TIDE score indicated poor response to ICI, our study revealed that the TIDE score was significantly decreased in the low-risk group, which was consistent with our hypothesis. Therefore, HRL signature might facilitate the application of ICI for the treatment of glioma. In IMvigor210 cohort, HRL signature was significantly associated with the prognosis of urothelial carcinoma patients, and high risk score indicated favorable prognosis. According to our results, the high-risk group had high immune infiltration and well responses to immunotherapy, which might account for the favorable prognosis of high-risk group in IMvigor210 cohort.

Recently, immune microenvironment was shown to play a critical role in cancer development (37–40). Multiple studies have demonstrated the immunosuppressive context surrounding glioma cell (41). Glioma cells would promote the expression of immune checkpoints such as programmed cell death 1 ligand (PD-L1) to induce immune escape (42). Besides, glioma cells would activate tumor-associated macrophages and regulatory T (Treg) cells, which suppressed the activities of cytotoxic T cells (43). HIF-1α was reported to regulate the functions and differentiations of myeloid-derived suppressor cells (44), which were a major component of immune-suppressive network. Besides, HIF-1α could increase the expression of PD-L1 by binding to its hypoxia response elements (45). Therefore, hypoxia microenvironment would mediate immune-suppressive effects and facilitate the progression of tumor cells. In this study, we found that high-risk group was associated with HIF-1α and immune-related pathways. Genes highly expressed in high-risk group was enriched in hypoxia and immune processes. Besides, high-risk group had a high infiltration of dendritic cell, macrophage, and T cells, which might be due to its hypoxia characteristics. However, it should be noted that the direct effect of HRLs on immune infiltration required additional experiments, and high immune infiltration was a characteristic of high-risk group rather than a result caused by HRLs. It seemed to be contradictory that the high immune infiltration indicated favorable prognosis in head and neck cancer whereas it indicated poor prognosis in LGG (18, 25, 26). Nevertheless, high infiltration of cytotoxic immune cells could suppress the development of tumor cells and result in favorable prognosis. In contrast, high infiltration of immunosuppressive immune cells such as M2 subtype macrophage and myeloid-derived suppressor cell would promote the progression of tumor and lead to poor prognosis. Therefore, immune-activating strategies such as ICIs remain to be promising therapeutics for glioma.

A previous study reported that immune infiltration-related lncRNA signature could predict responses to ICIs in non-small cell lung cancer patients, indicating the potential crosstalk between immune infiltration and immunotherapy response (46). Jiang et al. suggested that CTL-high tumors tended to evade from immune surveillance through inducing T cell dysfunction, which was defined as “non-responders” in TIDE algorithm (20). In our study, ssGSEA algorithm revealed that activated CD8+ T cell was highly enriched in high-risk group, which indicated a poor response to ICIs and was consistent with the elevated TIDE score in high-risk group.

Numerous studies have reported the role of lncRNA as biomarkers or potential therapeutic targets in cancers and other diseases (47, 48). Signatures composed on lncRNAs also exhibited promising value in predicting the prognosis and recurrence of cancers. Zhou et al. identified a six-lncRNA signature that could efficiently predict the risk of tumor recurrence in patients with colon cancer (49). Besides, signatures composed of immune-related lncRNA was shown to indicate the prognosis of patients with hepatocellular carcinoma, breast cancer, lung adenocarcinoma, and esophageal squamous cell carcinoma (50–53). In glioma, lncRNA signature was implicated to be a promising biomarker of tumor progression (54). However, to our limited knowledge, no study had reported the role of HRLs in the prognosis and immune microenvironment of gliomas. Our study constructed a 19-HRL signature that was associated with the prognosis the LGG patients. Besides, two major HRLs of the risk signature, LINC00941 and BASP1-AS1, were selected as the representation of HRL signature. Bioinformatic analyses indicated that LINC00941 and BASP1-AS1 were significantly associated with the prognosis and immune infiltration in LGG. *In vitro* experiments revealed that the inhibition of LINC00941 could significantly suppress the proliferation of glioma cells, whereas the inhibition of BASP1-AS1 exerted reversed effects. Previous studies indicated that ion channel was associated with the progression and prognosis of glioma (55, 56). Moreover, cell senescence was reported to correlate with the proliferation and migration of glioma (57, 58). Through the construction of ceRNA network, we found that LINC00941 and BASP1-AS1 might affect the proliferation of glioma cells *via* regulating ion channel activities and cell senescence. In addition, in high-LINC00941 and low-BASP1-AS1 group, EMT and TNF-α signaling pathways were highly enriched. Since EMT and TNF-α were found to be highly involved in the progression of glioma (59, 60), the increased level of LINC00941 and the decreased level of BASP1-AS1 might account for the promoted proliferation of glioma cells through activating EMT and TNF-α signaling pathway. Therefore, LINC00941 and BASP1-AS1 could be potential targets for glioma treatment.

## Conclusions

To sum up, our study constructed a novel HRL signature that could predict the prognosis and was associated with immune infiltration of LGG. HRLs could be novel biomarkers to predict the prognosis and potential targets for LGG treatment.

## Data Availability Statement

The original contributions presented in the study are included in the article/**Supplementary Material**. Further inquiries can be directed to the corresponding authors.

## Author Contributions

QC and CL conceived, designed, and supervised the study. SX drafted the manuscript. SX, LT, and ZL collected the data. SX performed data analysis and visualization. All authors contributed to the article and approved the submitted version.

## Funding

This work was supported by National Natural Science Foundation of China (81703622, 81873635, 81902553, 82073893), Natural Science Foundation of Hunan Province (2018JJ3838, 2019JJ50942), Key Research and Development Program of Hunan Province (2018SK2101), China Postdoctoral Science Foundation (2018M633002, 2021T140750), Hunan Provincial Health Committee Foundation of China (C2019186), and Xiangya Hospital Central South University postdoctoral foundation.

## Conflict of Interest

The authors declare that the research was conducted in the absence of any commercial or financial relationships that could be construed as a potential conflict of interest.

## Publisher’s Note

All claims expressed in this article are solely those of the authors and do not necessarily represent those of their affiliated organizations, or those of the publisher, the editors and the reviewers. Any product that may be evaluated in this article, or claim that may be made by its manufacturer, is not guaranteed or endorsed by the publisher.

## Supplementary Material

Supplementary Figure S1 Kaplan-Meier analysis of randomly selected eight HRLs in LGG patients.

Supplementary Figure S2 The correlation between risk score and hypoxia-related genes.

Supplementary Figure S3 Volcano plot of differentially expressed genes. **(A, B)** Differentially expressed genes between high-risk and low-risk groups in the TCGA **(A)** and CGGA **(B)** datasets. **(C, D)** Differentially expressed genes between high or low expression of LINC00941 **(C)** and BASP1-AS1 **(D)** groups.

Supplementary Figure S4 Kaplan-Meier analysis of GNAI1 and PGAM1 in TCGA dataset.
