# Supplementary figures and images for: Hypoxia-Related lncRNA Correlates With Prognosis and Immune Microenvironment in Lower-Grade Glioma
Source: Front Immunol. 2021 Sep 30;12:731048. doi: 10.3389/fimmu.2021.731048 (PMC8514865; doi:10.3389/fimmu.2021.731048)

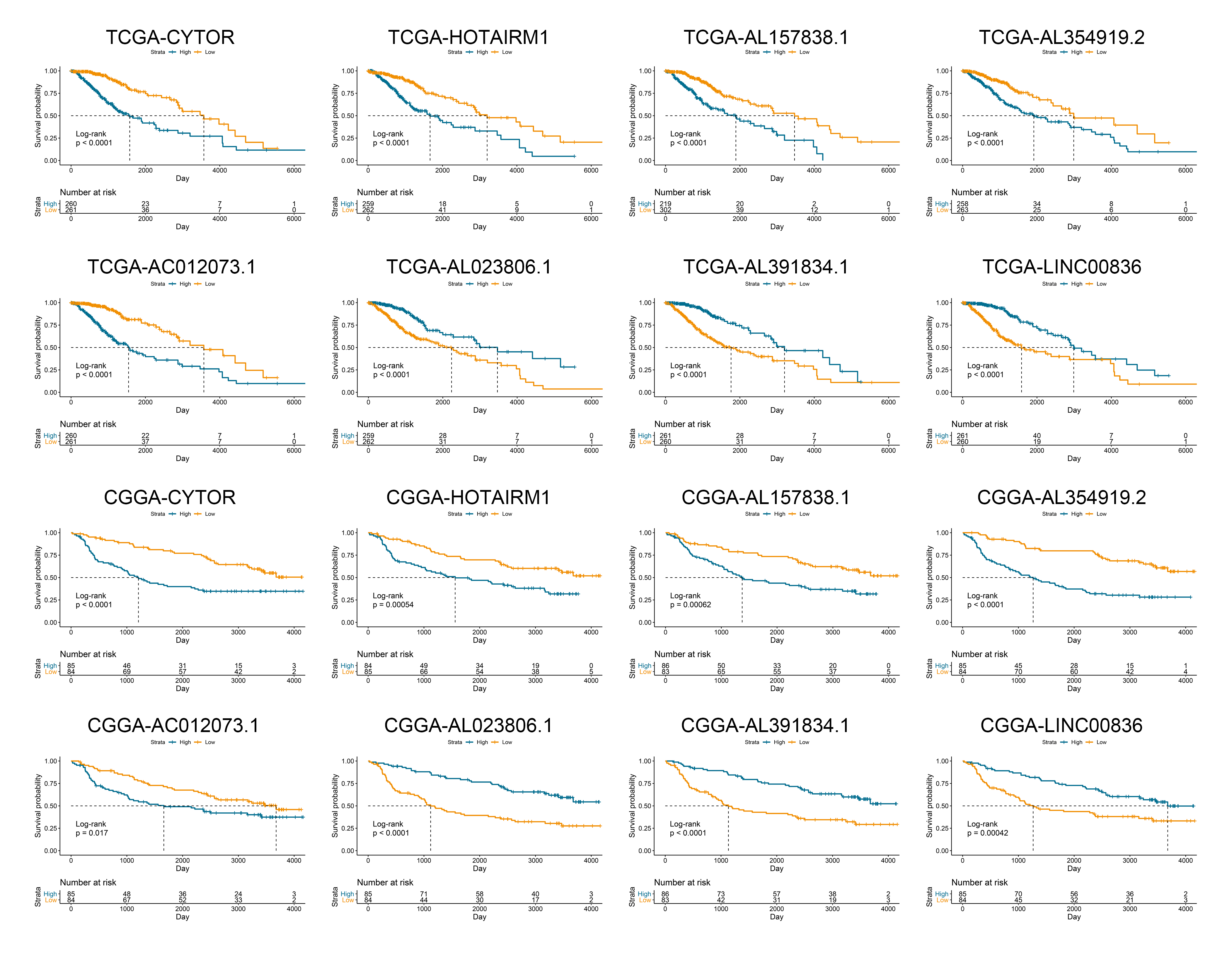

Supplement: Supplementary Figure S1 — Kaplan-Meier analysis of randomly selected eight HRLs in LGG patients. [file Image_1.tif]

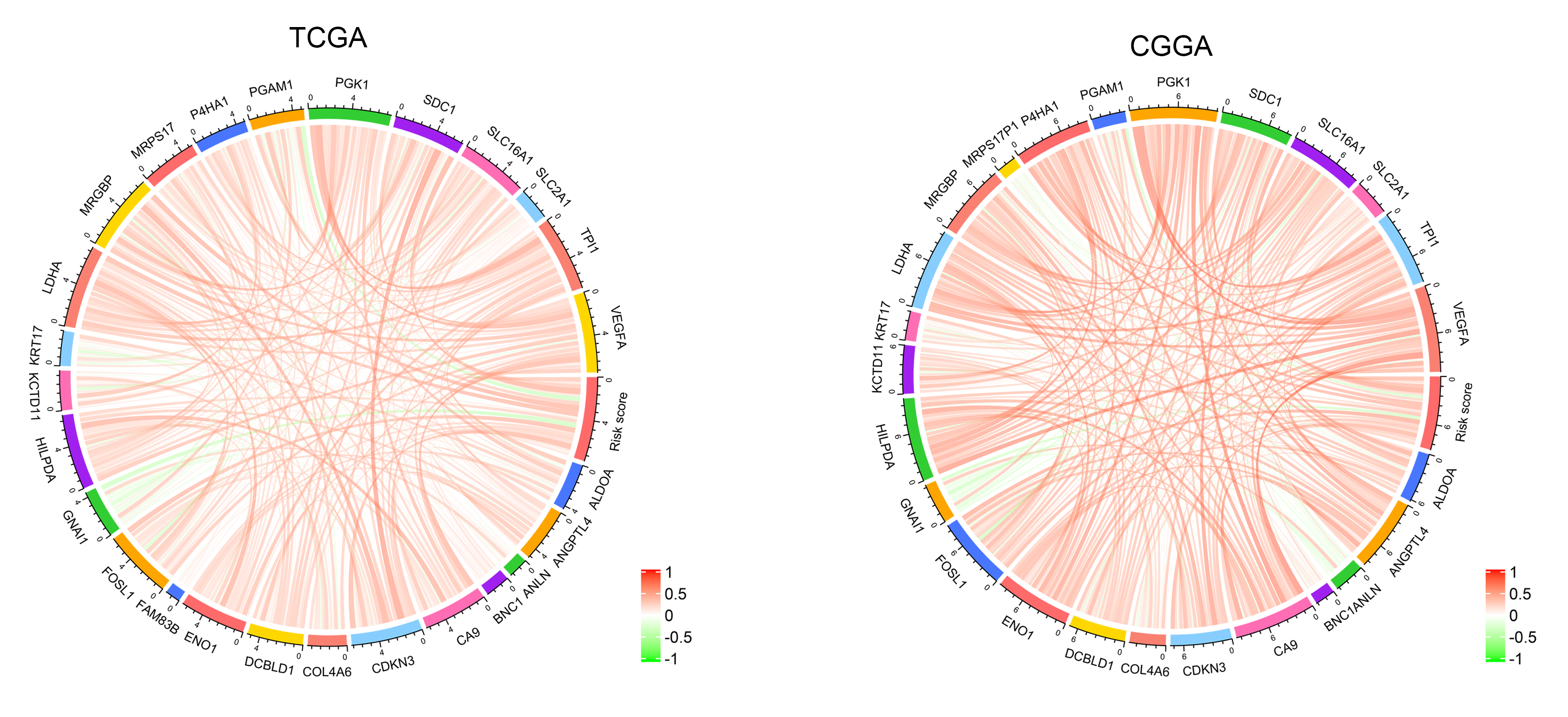

Supplement: Supplementary Figure S2 — The correlation between risk score and hypoxia-related genes. [file Image_2.tif]

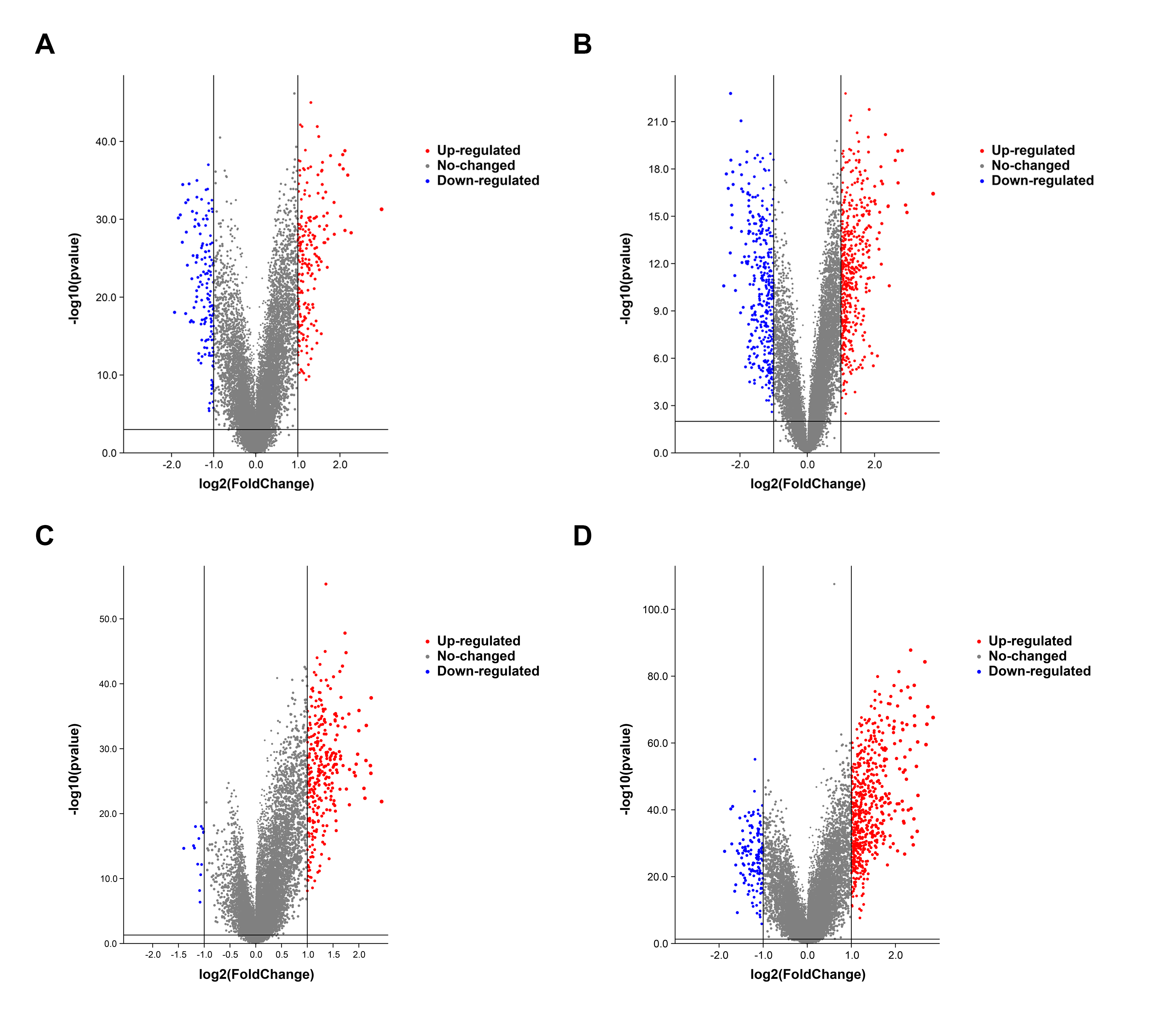

Supplement: Supplementary Figure S3 — Volcano plot of differentially expressed genes. (A, B) Differentially expressed genes between high-risk and low-risk groups in the TCGA (A) and CGGA (B) datasets. (C, D) Differentially expressed genes between high or low expression of LINC00941 (C) and BASP1-AS1 (D) groups. [file Image_3.tif]

# GNAI1

Strata + High + Low

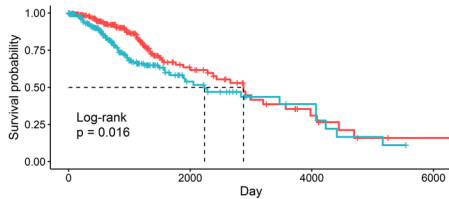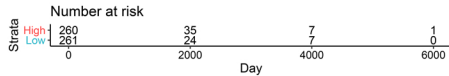

# PGAM1

Strata + High + Low

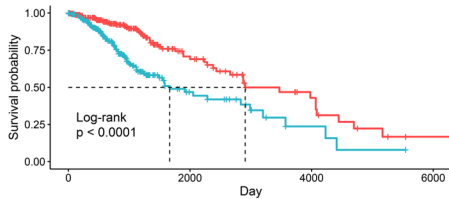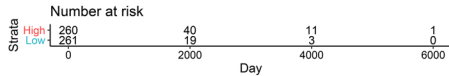

Supplement: Supplementary Figure S4 — Kaplan-Meier analysis of GNAI1 and PGAM1 in TCGA dataset. [file Image_4.pdf]
